# The differences between carotid web and carotid web with plaque: based on multimodal ultrasonic and clinical characteristics

**DOI:** 10.1186/s13244-024-01650-7

**Published:** 2024-03-18

**Authors:** Chao Hou, Shuo Li, Lei Zhang, Wei Zhang, Wen He

**Affiliations:** 1 Department of Ultrasound, Lanzhou University Second Hospital, No.82 Cuiyingmen, Chengguan District, Lanzhou, Gansu Province 730030 China; 2 Department of Ultrasound, The Affiliated Hospital of Southwest Medical University, No. 25 TaiPing St, Jiangyang District, Luzhou, Sichuan 646000 China; 3 Department of Ultrasound, Beijing Tiantan Hospital, Capital Medical University, No.119, South Forth Ring Road West, Fengtai District, Beijing, 100070 China

**Keywords:** Angle, Carotid web, Plaque, Ultrasonography

## Abstract

**Objective:**

This study aimed to examine the clinical and multimodal ultrasonic characteristics differences between carotid web (CW) and CW with plaque as well as the potential risk factors for stroke caused by CW.

**Methods:**

We retrospectively enrolled patients diagnosed with CW by CTA or high-resolution MRI (HRMRI) and simultaneously underwent contrast enhanced ultrasound (CEUS) and superb microvascular imaging examinations from January 2015 to October 2022. The CW angle was measured using computer-aided software. The variations between CW and CW with plaque were evaluated, and univariable and multivariable logistic regressions were utilized to identify possible risk predictors for stroke caused by CW.

**Results:**

Two hundred ninety-nine patients with an average age of 60.85 (± 8.77) years were included. Sex, age, history of smoking, alcohol, hypertension, diabetes mellitus, homocysteine level, and treatment, as well as web length and thickness, luminal stenosis, location wall, number, CW angle, and CEUS enhancement, were quite different among CW and CW with plaque patients (*p* < 0.05). The logistic regression analysis showed that web length was an independent predictor of luminal stenosis in CW patients. For patients with CW and plaque, plaque and web thickness, as well as plaque enhancement, were associated with stenosis. Furthermore, luminal stenosis and plaque length were risk factors for symptoms.

**Conclusion:**

The multimodal ultrasonic and clinical manifestations of CW and CW with plaque are quite different. Web length is an independent risk factor for carotid artery stenosis in CW patients, whereas luminal stenosis and plaque length were risk factors for symptoms in CW with plaque patients.

**Critical relevance statement:**

Exploring the similarities and differences between the carotid web and the carotid web with plaque, based on the stereo-geometric spatial position relationship and hemodynamic changes, may provide further insights into the underlying mechanisms of stroke occurrence caused by the carotid web.

**Key points:**

1. Multimodal ultrasonic and clinical manifestations of carotid web and carotid web with plaque are substantially different.

2. A thin triangular endoluminal defect is identified as a typical feature of the web on superb microvascular imaging, and two kinds of typical ultrasonic features of CW with plaque are also identified.

3. Web length is an independent risk factor for carotid stenosis in carotid web patients, whereas luminal stenosis and plaque length are risk factors for symptoms in patients with CW and plaque.

**Graphical Abstract:**

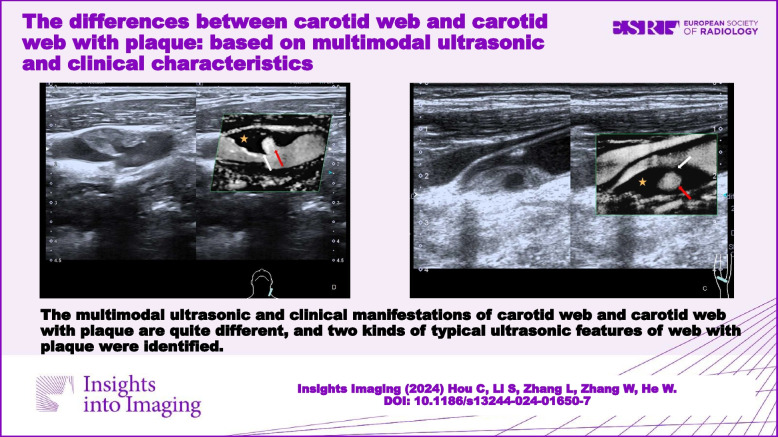

**Supplementary Information:**

The online version contains supplementary material available at 10.1186/s13244-024-01650-7.

## Introduction

Stroke is a grave disease that poses a significant threat to human health and ranks as the second leading cause of death globally, following ischemic heart disease [1]. Among the various types of strokes, cryptogenic ischemic stroke is particularly challenging for stroke specialists, accounting for approximately 26% of all ischemic strokes [2]. Carotid web (CW), a cause of cryptogenic stroke (2.5–37%) [3–5] with an undetermined etiology and epidemiology, has a high stroke recurrence rate (11.4–27.3%) [5, 6] under conservative medical therapy.

CW is a kind of intimal fibromuscular dysplasia characterized by a shelf-like projection of intimal fibrous tissue into the carotid bulb [7]. It was first reported by Rainer as a cause of recurrent left hemispheric events in an otherwise healthy young individual [8]. Currently, there are approximately 90 reports in the global literature regarding CW. The diagnosis of CW is primarily based on typical imaging findings and the exclusion of other lesions in the same location (such as arterial dissection, non-calcified atherosclerotic plaque, and intraluminal thrombus). Although several cases of plaque or thrombus accompanied by CW have been documented [9, 10], there is a lack of systematic exploration of the clinical and ultrasound imaging manifestations of CW with plaque. Studies have shown that both contrast-enhanced ultrasound (CEUS) and superb microvascular imaging (SMI) are valuable imaging methods for identifying plaque vulnerability [11]. However, it remains to be studied whether these two new technologies add additional value to the traditional ultrasound examination of CW and CW with plaques. In addition, some CWs were missed during the initial imaging examination [5, 10, 12], so sonographers should be familiar with the multimodal sonography features and clinical characteristics of CW and CW with plaques in order to reduce misdiagnosis and missed diagnosis. This will ultimately improve clinical intervention and treatment. Therefore, the goal of this study is to analyze the multimodal ultrasonic and clinical differences between CW and CW with plaque as well as identify potential risk factors for strokes associated with CW.

## Methods

### Study population and design

This study retrospectively analyzed all patients who underwent carotid artery CEUS and SMI examinations at our hospital from January 2015 to October 2022. The patients were categorized based on different reasons for examinations, including stroke, transient ischemic attack, suspected CW, or suspected vulnerable carotid artery plaque. The inclusion criteria for the study were patients aged 18 years or older with complete imaging and clinical data. CW was diagnosed using computed tomographic angiography (CTA) and/or high-resolution magnetic resonance imaging (HRMRI) with specific criteria: a thin and linear filling defect along the wall of the carotid bulb on oblique sagittal CTA/HRMRI visible as a septum on the axial section [4, 13, 14]. This diagnosis could be made with or without carotid plaque. A plaque was defined as inner-medial membrane thickening greater than 1.5 mm or localized thickening of more than half of the surrounding tissue. The exclusion criteria included incomplete medical history, poor quality of ultrasound images, history of carotid endarterectomy (CAE) or stent (CAS) placement, lack of CTA and/or HRMRI images, severe renal disease, and cerebrovascular events caused by other diseases such as carotid dissection, vasculitis, or atrial fibrillation.

Baseline characteristics and associated risk factors were collected, including sex, age, body mass index (BMI), smoking history, alcohol consumption, hypertension, diabetes mellitus, clinical symptoms, and clinical management (CAE, CAS, or conservative treatment). Additionally, levels of total cholesterol (TC), triglyceride (TG), low-density lipoprotein (LDL), high-density lipoprotein (HDL), and homocysteine (Hcy) were recorded. Symptomatic refers to the occurrence of a transient ischemic attack, stroke, or transient amaurosis on the ipsilateral side of the CW within the past 6 months. The clinical symptoms were evaluated by an experienced neurologist. For those who opted for conservative treatment, a follow-up was conducted by reviewing their medical records or through phone communication with the patients (or their relatives, in cases where the patients had cognitive or language impairments). The end event was the occurrence of cerebrovascular events.

### Ultrasound imaging

Ultrasound equipment (Aplio 900, Canon Medical Systems Corporation, Japan; Aplio 500, Toshiba Medical Systems Corporation, Japan; Aixplorer, Supersonic Imagine, France) with high-frequency (5–14 MHz) linear array probes was used. The following parameters were obtained at baseline using a multimodal ultrasound protocol: echo, length, thickness, number (single or multiple), location (right or left artery), arterial wall (anterior, posterior, or side wall), degree of carotid stenosis, and the size of plaque or thrombosis if the CW is combined with them. Color Doppler flow imaging (CDFI) was then utilized to observe the presence of local turbulence and swirling blood flow. The evaluation of carotid stenosis was conducted using grayscale and CDFI criteria from the 2003 Society of Radiologists in Ultrasound Consensus Conference [15]. The CW angle in each case was calculated using open-source computer-aided software (Adobe Photoshop, https://www.adobe.com/). The CW angle refers to the angle between the central line of the carotid artery and the tangent line [16].

SMI was performed to detect the surface morphology of stenosis and the morphological characteristics of CWs. Carotid CEUS was utilized to observe the morphological changes and enhancement (details of the SMI and CEUS settings were available in Supplementary 1). The enhancement intensity was visually classified into three grades according to the grading criteria from a previous study [17]: grade 0: no visible microbubbles; grade 1: moderate microbubbles confined to the shoulder and/or adventitial side; grade 2: extensive intraplaque enhancement. All ultrasound images were reviewed by two radiologists (S.L. and L.Z.) with over 10 years of experience in vascular ultrasound using a double-blind method. Any disagreements were resolved through consensus.

The CTA/HRMRI imaging scanning protocols were the same as in the prior study [18], and the details of protocols are available in Supplementary 1. All CTA and HRMRI images were reviewed by two experienced radiologists (W.Z. and W.H.).

### Statistical analysis

The measurement data that conformed to a normal distribution were presented as mean ± standard deviation (SD) or median, while the discrete variables were described as percentages. To ensure an adequate sample size, individual missing values were replaced with the average of adjacent values rather than deleting them. Differences between groups were compared using an independent sample *t*-test or chi-square test. Variables with a significant trend (*p* < 0.1) in the univariate logistic analysis were included in the multivariable logistic model and backward eliminated to a significance level of 0.05. To test interobserver agreement for CW angle measurement, a random sample of 30 cases was reanalyzed by the same author after a 1-month interval. Interobserver agreement was determined using K analysis. *p* < 0.05 was considered statistically significant. All statistical analyses were performed using SPSS version 25.0 (IBM, Chicago, IL, USA).

## Results

### Clinical characteristics

During the 8-year period, a total of 1422 patients underwent CEUS and SMI examinations. Among them, 358 patients were initially suspected to have CW based on ultrasound. After applying inclusion and exclusion criteria, a final sample of 299 patients with CW (mean age ± SD, 60.85 ± 8.77 years; 236 men, 78.9%) diagnosed by CTA/HRMRI were included in the study. The flowchart diagram of patient selection was shown in Supplementary Fig. 1. Out of the 299 participants, 68 had CWs, while 231 had CW with plaque formation. Among the included individuals, 47.2% (141/299) had arterial stenosis greater than 50%, and 27.8% (83/299) were symptomatic. It is worth mentioning that among the CW group, 5.88% (4/68) of patients with thrombosis were symptomatic (100%), while in the CW with plaque group, 8.2% (19/231) of patients developed concomitant thrombosis, with 10 of them (52.6%) exhibiting symptoms. Significant differences were observed between the two groups in terms of sex, age, history of smoking, alcohol consumption, hypertension, diabetes mellitus, and the level of Hcy (*p* < 0.05). However, no significant differences were found in other indicators, including BMI, symptoms, and the levels of TG, TC, HDL, and LDL (*p* > 0.05) (Table 1).

**Table 1:** Comparison of patients with baseline information

Parameters	Total (*n* = 299)	CW (*n* = 68)	CW with plaque (*n* = 231)	*t*/*χ*^2^	*p* value
Male, *n* (%)	236 (78.9)	29 (42.6)	207 (89.6)	69.672	< 0.001
Age (years)	60.85 ± 8.77	50.61 ± 12.49	63.87 ± 7.65	-10.664	< 0.001
BMI (kg/m^2^)	24.61 ± 3.62	25.23 ± 3.09	24.57 ± 0.17	0.963	0.337
Smoking, *n* (%)	184 (61.5)	15 (22.1)	179 (73.2)	57.962	< 0.001
Drink, *n* (%)	168 (56.2)	26 (38.2)	142 (61.5)	11.523	0.001
Hypertension, *n* (%)	206 (68.9)	20 (29.4)	186 (80.5)	64.035	< 0.001
Diabetes mellitus, *n* (%)	140 (46.8)	12 (17.6)	128 (55.4)	30.09	< 0.001
Hcy (mmol/L)	16.07 ± 8.45	13.12 ± 4.52	16.26 ± 8.61	-2.421	0.025
TG (mmol/L)	1.32 ± 0.60	1.40 ± 0.69	1.31 ± 0.68	0.454	0.651
TC (mmol/L)	3.89 ± 0.84	4.12 ± 0.97	3.88 ± 0.95	0.926	0.356
HDL (mmol/L)	1.19 ± 0.27	1.25 ± 0.22	1.19 ± 0.31	0.82	0.413
LDL (mmol/L)	2.24 ± 0.72	2.37 ± 0.87	2.23 ± 0.81	0.634	0.527
Symptom, *n* (%)	87 (29.09)	15 (22.1)	72 (31.2)	2.113	0.172
Treatment, *n* (%)				25.759	< 0.001
CAE	72 (24.1)	4 (6.0)	68 (29.3)		
CAS	10 (3.3)	0	10 (4.3)		
Conservative therapy	217 (72.6)	64 (94.0)	153 (66.4)		
Follow-up, *n* (%)				0	1.00
Cerebrovascular events	7 (4.0)	2 (3.6)	5 (4.1)		
No cerebrovascular events	170 (96.0)	53 (96.4)	117 (95.9)		

*CW* Carotid web, *BMI* Body mass index, *Hcy* Homocysteine, *TG* Triglyceride, *TC* Total cholesterol, *LDL* Low density lipoprotein, *HDL* High-density lipoprotein, *CAE* Carotid endarterectomy, *CAS* Carotid stenting

Four (5.97%) CW patients and 68 (29.3%) CW with plaque patients received CAE. Representative surgical specimens were available in Supplementary Fig. 2A–B). And 10 (4.31%) patients with CW and plaque underwent CAS. After a median follow-up of 24 months (ranging from 3 to 62), outcomes were observed for 217 patients who received conservative therapy. Among these patients, 4 died (1 in the CW group and 3 in the CW with plaque group), 36 were lost to follow-up without any available records (8 in the CW group and 28 in the CW with plaque group), and 7 (4.0%) patients experienced cerebrovascular events. These events included 2 in the CW group, both of whom had previous stroke experience, and 5 in the CW with plaque group, 2 of whom had previous stroke experience (Table 1).

### Ultrasound characteristics

**Fig. 1:**
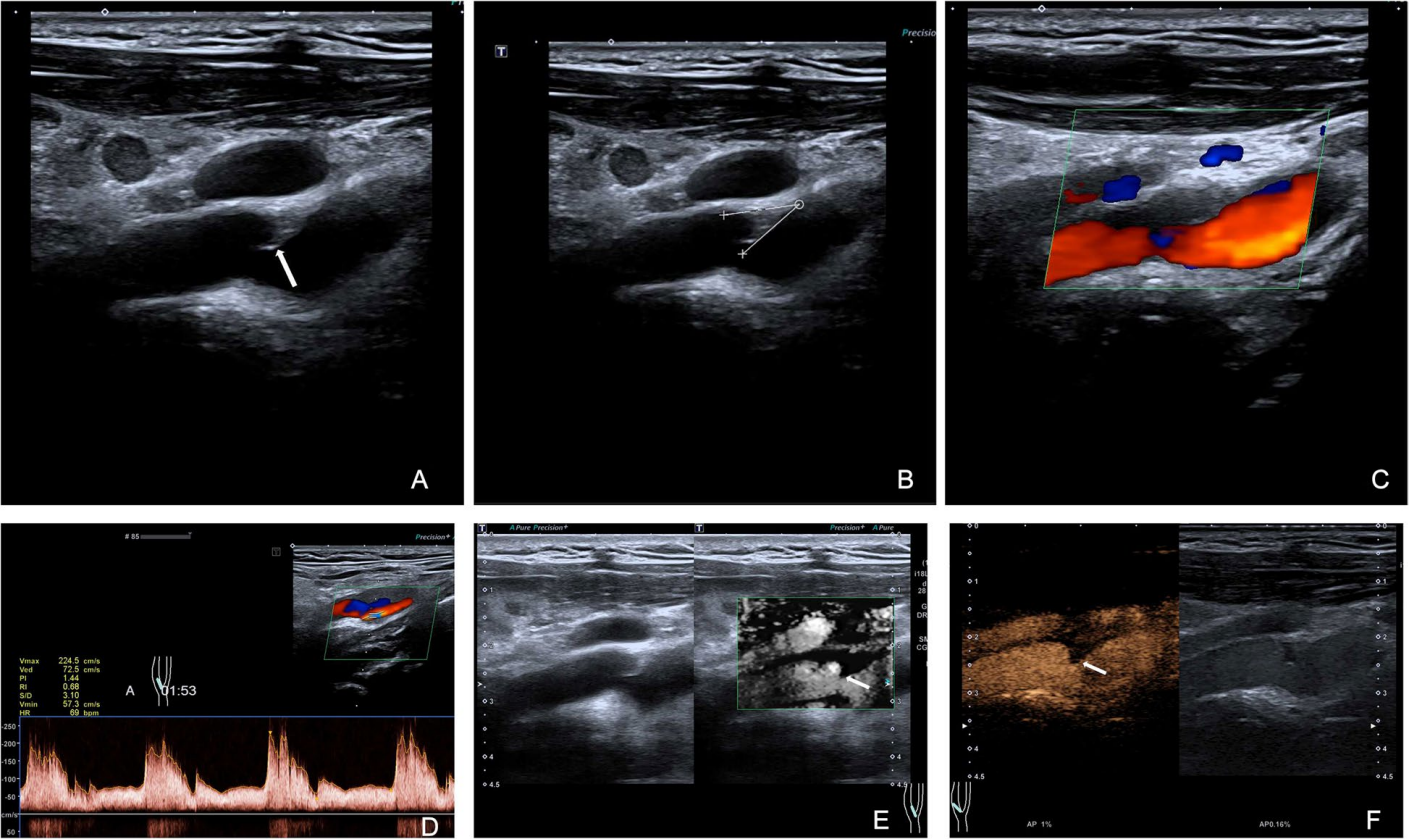
Multimodal ultrasound features of a carotid web (white arrow) locates on the anterior wall of the right carotid artery bifurcation are presented. **A** An isoechoic shelf-like structure protrudes into the lumen. **B** The carotid web angle (31.3°) is measured using computer-aided software. **C** Color doppler ultrasound reveals swirling blood around the web. **D** The carotid web causes moderate luminal stenosis. **E** Superb microvascular imaging reveals a triangular endoluminal defect on the longitudinal plane. **F** Contrast-enhanced ultrasound shows no enhancement of the web

Upon conventional ultrasonography, the CW emerged as an iso- or hypoechoic shelf-like structure protruding into the lumen (Fig. 1A–F, Supplementary Fig. 3). There were significant differences between the two groups in terms of web length and thickness, luminal stenosis, arterial wall location, number of webs, CW angle, and CEUS enhancement (*p* < 0.05) (Table 2). The mean CW angle in CW patients was 37.33 (± 23.14) °, while in CW with plaque patients, it was 58.72 (± 36.95) °. The Kappa value for interobserver agreement of the CW angle was 0.85, indicating excellent agreement.

**Table 2:** Comparison of patients with ultrasonic features

Parameters	Total (*n* = 299)	CW (*n* = 68)	CW with plaque (*n* = 231)	*t*/χ^2^	*p* value
Web number, *n* (%)				8.465	0.003
Single	246 (82.3)	64 (94.1)	182 (78.8)		
Multiple	53 (17.7)	4 (5.9)	49 (21.2)		
Web length (mm)	6.51 ± 2.82	4.99 ± 2.22	7.00 ± 2.82	-5.307	< 0.001
Web thickness (mm)	1.55 ± 0.73	0.95 ± 0.43	1.23 ± 0.79	-2.702	0.007
Web location, *n* (%)				1.346	0.271
Right artery	146 (48.8)	29 (42.6)	117 (50.6)		
Left artery	153 (51.2)	39 (54.4)	115 (49.6)		
Arterial wall, *n* (%)				28.344	< 0.001
Anterior	71 (23.7)	4 (5.9)	67 (29.0)		
Posterior	186 (62.2)	61 (89.7)	125 (54.1)		
Sides	42 (14.0)	3 (4.4)	39 (16.9)		
Web angle (°)	52.42 ± 34.83	37.33 ± 23.14	58.72 ± 36.95	-4.255	< 0.001
Plaque length (mm)	/	/	18.41 ± 7.39^a^		
Plaque thickness (mm)	/	/	3.91 ± 1.33		
≥ 50% stenosis, *n* (%)	141 (47.2)	7 (10.3%)	134 (58.0%)	47.997	< 0.001
CEUS enhancement, *n* (%)				129.48	< 0.001
Grade 0	121 (40.5)	68 (100)	53 (22.9)		
Grade 1	87 (29.1)	0	87 (33.7)		
Grade 2	91 (30.4)	0	91 (39.4)		

*CW* Carotid web, *CEUS* Contrast-enhanced ultrasound

^a^Missing value (*n* = 4)

**Fig. 2:**
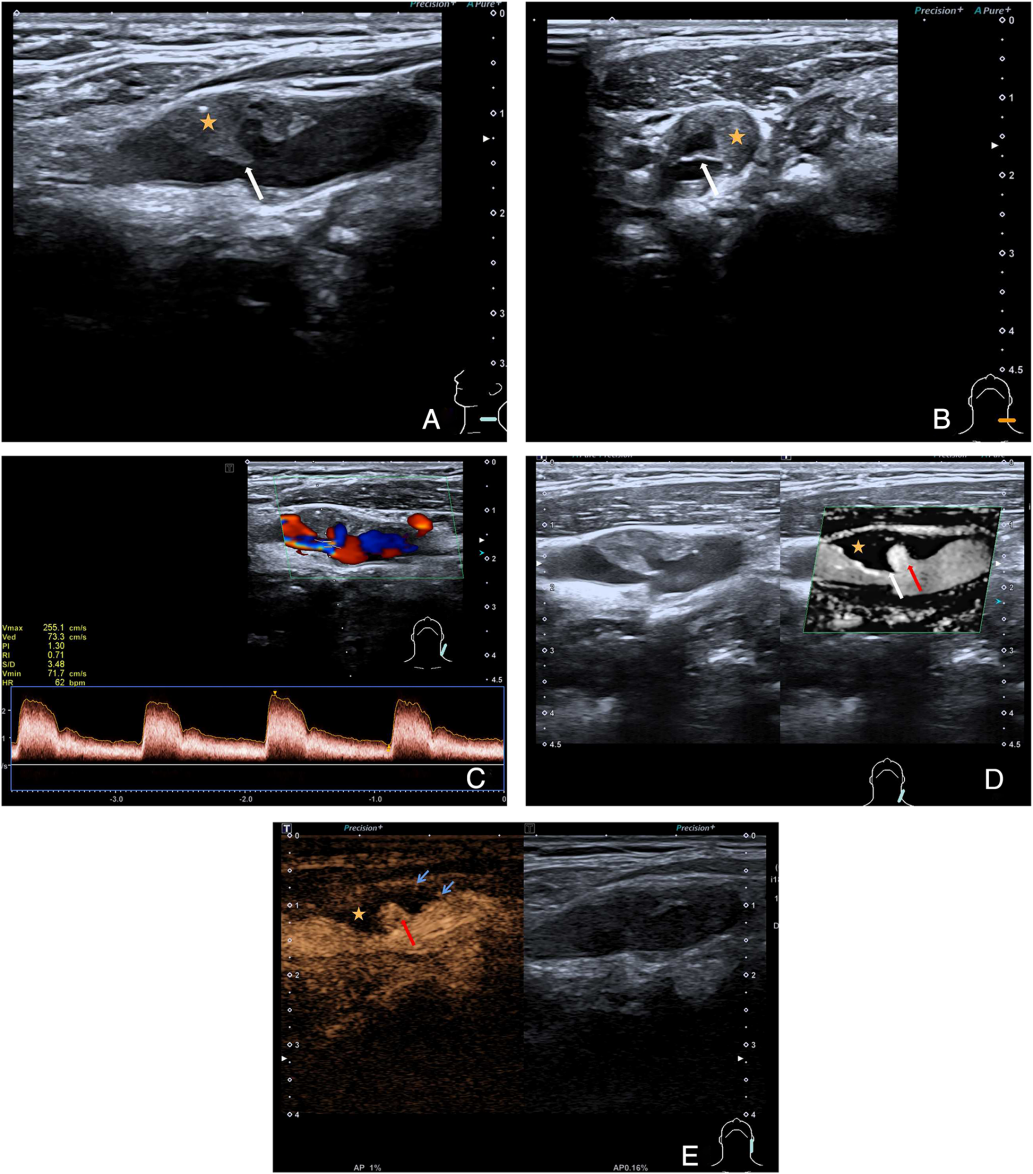
Multimodal ultrasound features of a carotid web (white arrow) with plaque formation (orange star) which locates on the anterior wall of the left carotid artery bifurcation. **A** B-mode ultrasound shows an uneven isoechoic plaque on the longitudinal plane with a shelf-like structure protruding into the lumen. **B** A thin membrane-like structure on the cross plane. **C** Color doppler ultrasound shows swirling blood and severe arterial stenosis. **D** Superb microvascular imaging reveals triangular endoluminal defect. **E** Contrast-enhanced ultrasound shows contrast agents entering the cavity (red arrow), with plaque enhancement (grade 1, blue arrows)

**Fig. 3:**
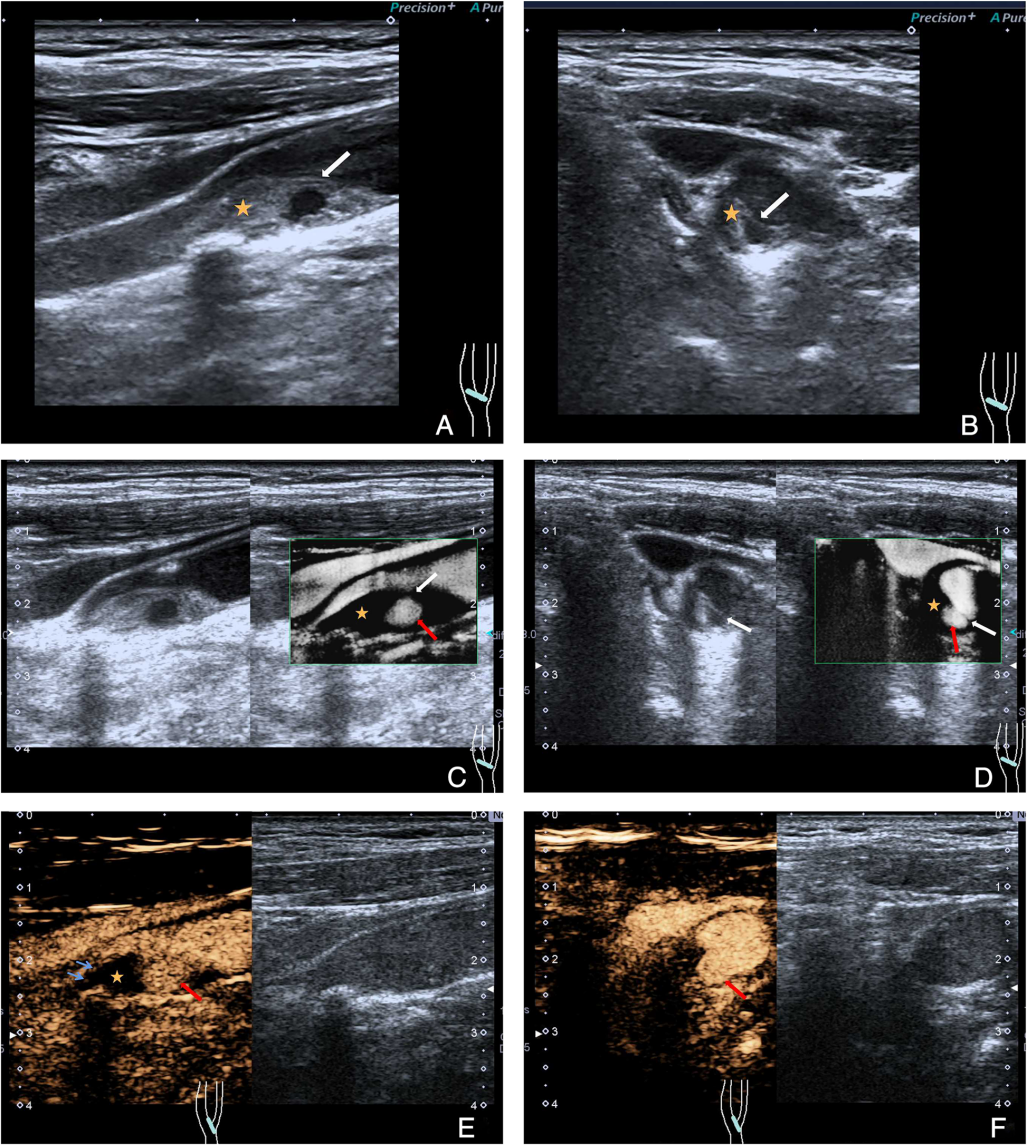
Multimodal ultrasound features of a carotid web (white arrow) grows along the long axis of the plaque (orange star) which locates on the posterior wall of the right carotid artery bifurcation. **A**, **B** B-mode ultrasound shows a mixed isoechoic plaque with a membrane-like structure and a cavity. **C**, **D** Superb microvascular imaging reveals cliff-like defect on the longitudinal plane and liner defect on the cross-section. **E**, **F** Contrast-enhanced ultrasound shows contrast agents entering the cavity (red arrow), with plaque enhancement (grade 1, blue arrows)

On CEUS, all CWs showed perfusion deficiency with no enhancement. However, in the CW with plaque group, the percentages of plaque enhancement were as follows: grade 0: 22.9% (53/231), grade 1: 37.7% (87/231), and grade 2: 39.4% (91/231). On SMI, most CWs appeared as triangular endoluminal defects or wedge-like structures on the longitudinal plane but as cliff-like filling defects on the axil plane (Fig. 2A–E, Supplementary Fig. 4, Supplementary Movies 1, 2 and 3). The triangular endoluminal flaw was found in 97.1% (66/68) of CWs and in 72.7% (168/231) of CWs with plaques. To be noted, there was another type of CW with plaque, in which both CDFI and CEUS showed a swirling pattern of blood flow, and contrast agents were able to penetrate the cavity. SMI, on the other hand, revealed a transversal linear defect on the cross-section, but no triangular flaw was observed within the inner lining along the longitudinal plane (Fig. 3A–F, Supplementary Fig. 5).

### Logistic regression analysis

We conducted univariate and multivariate logistic regression analyses to investigate potential risk factors for symptoms and luminal stenosis. In the CW group, logistic analyses indicated that only web length was associated with luminal stenosis (OR = 1.653, 95% CI 1.139–2.399, *p* = 0.008). In the CW with plaque group, logistic analysis revealed that web thickness (OR = 1.87, 95% CI 1.099, 3.182, *p* = 0.021), plaque thickness (OR = 1.587, 95% CI 1.199–2.101, *p* = 0.001), and CEUS enhancement (OR = 0.37, 95% CI 0.177–0.773, *p* = 0.008) were predictors of luminal stenosis. Additionally, luminal stenosis (OR = 1.86, 95% CI 1.021–3.391, *p* = 0.043) and plaque length (OR = 0.955, 95% CI 0.918–1.014, *p* = 0.023) were identified as risk factors for symptoms (Supplementary Tables 1 and 2).

## Discussion

Angiographically, the CW is defined as a thin intraluminal filling defect located at the posterior wall of the internal carotid arterial bulb [19, 20]. In our study, the majority of CWs (62.2%) were found in the posterior wall, while some were present in the anterior and side walls. The overall prevalence of CW was 21.0% (299/1422), and out of these CW patients, 27.8% (83/299) experienced symptoms, which is higher than what previous studies have reported [5, 13]. The elevated prevalence of CW can be attributed to several factors. Firstly, our institution is a renowned national neurology research center in China, attracting patients from all over the country who have suspected neurological symptoms and seek medical attention. In addition, our study included individuals who had undergone both carotid CEUS and SWI over an 8-year period, resulting in a narrower inclusion criteria and consequently an increased detection rate.

Consistent with previous studies [14, 18, 21, 22], our results indicated that gender and age differences in the two groups were statistically significant. In the CW group, 57.4% of the participants were women with an average age of 50.61 (± 12.49) years. On the other hand, in the CW with plaque group, only 10.4% were women, with an average age of 63.87 (± 7.65) years. Abnormal hormone levels, potentially caused by endogenous or exogenous estrogen, may contribute to the higher prevalence of CW in women. This hypothesis is supported by a systematic review which found that a majority of female patients had a history of oral contraceptive use [22]. Additionally, plaque formation is a chronic disease that is related to smoking, alcohol, hypertension, and diabetes mellitus; therefore, CW with plaque is more prone to occur in the elderly. Turbulence in the area beneath CW causes decreased blood flow velocity, leading to the formation of high wall shear stress (WSS) at the proximal end of the stenosis. Conversely, blood flow stagnation and low WSS occur at the distal part [23]. The presence of low WSS contributes to the transformation of endothelium cells and affects the inflammatory process [24, 25], ultimately leading to the initiation and progression of atherosclerosis [26]. This intricate process of CW-plaque formation also provides insights into why the disease tends to occur later in life compared to CW alone.

Multi-modular ultrasound assists to differentiate CW and CW with plaque formation. On conventional ultrasound, CW appears as a hypo- or isoechoic shelf-like structure without motion, protruding into the lumen of the carotid artery. In contrast, CW with plaque formation has two distinct ultrasound manifestations. Firstly, the web can grow in a cliff-like structure along the short axis of the plaque, protruding from its surface into the lumen. The web angle in this type can be either acute or obtuse. Secondly, webs can develop parallel to the plaque’s long axis, appearing as a membrane-like structure with a smooth, flat cavity. This contrasts with plaque ulceration, where the cavity lacks a complete membrane-like structure on the surface and usually has an uneven bottom [9, 10]. The formation of the cavity-like structure between the web and plaque is a result of webs that emerge from the vessel wall or plaque surface at various angles [27]. In this case, the CW angle exhibited a reduced magnitude, approaching 0°. CDFI can visualize changes in blood flow patterns, such as swirling blood flow, and estimate the severity of stenosis using hemodynamic parameters. The CEUS manifestations of CW and CW with plaque are quite different. The webs were non-enhancing with filling defects, whereas 87 plaques were classified as grade 1 and 91 plaques were classified as grade 2. The CEUS presentation of CW is associated with its pathological features, unlike plaques, where the main pathology of CW is intimal hyperplasia and fibroplasia [28, 29], while vulnerable plaques can be accompanied by neovascularization [17]. On SMI, thin triangular endoluminal flaws were observed as a specific feature in 97.1% of CW and in 72.7% of CW with plaque. However, in the second type of CW with plaque, a transversal linear defect was presented on the cross-section without the presence of a triangular flaw along the longitudinal plane.

Blood flow stagnation in the CW is believed to contribute to the development of thrombosis and recurrent strokes. The impact of circulatory disturbances on thrombosis has long been debated. High WSS and sudden changes in blood flow encourage platelet aggregation and vasoconstriction. Conversely, stagnation of blood flow and a reduction in WSS become more noticeable after stenosis occurs. Theoretically, a large CW angle generates more turbulence due to the streamlined shape of the artery being better suited to a small CW angle. However, both the CW angle and degree of stenosis affect the increased turbulence intensity (TI) on the downstream side of CW [16]. When the CW angle remains constant, the TI rises with an increase in the stenosis degree. Conversely, when the stenosis rate remains the same, the TI decreases as the CW angle increases [16]. Elevated TI can lead to blood stagnation, thereby raising the risk of thrombosis and causing an ischemic stroke. Our results showed that the web length was a risk factor for luminal stenosis in CW patients, but the correlation between luminal stenosis and symptoms, CW angle and symptoms, and angle and stenosis remains unclear. In fact, unlike the CW with plaque group, most symptomatic CW patients (80%, 12/15) had stenosis of less than 50%. These findings align with a meta-analysis conducted by Zhang [22]. Nevertheless, despite both CW and carotid plaque being stenotic lesions, their blood flow characteristics differ because of variations in the geometry of stenotic lesions [16]. Our study showed that the CW angle in the CW group was smaller than that in the CW with plaque group, and the rate of stenosis was lower; both of these differences were statistically significant, but the symptom difference between the two groups was not. In addition to the geometric differences, plaque eccentricity and ulceration can increase downstream turbulence [24]. Moreover, web and plaque thickness, as well as plaque enhancement, was correlated with stenosis in the CW with plaque group, whereas luminal stenosis and plaque length were risk factors for symptoms. These findings indirectly suggest that there are different underlying mechanisms of stroke in CW patients and CW with plaque patients. Therefore, in future research, the construction of animal CW models and CW with plaque models, as well as the exploration of the similarities and differences between the two based on the stereo-geometric spatial position relationship and hemodynamic changes [19, 30], may help further explain the mechanisms of stroke occurrence caused by CW.

Furthermore, it is claimed that carotid revascularization may be more effective than medical management alone in preventing stroke recurrence in patients with CW [14, 22]. Among these two groups, patients with CW were more likely to choose conservative therapy than patients with CW and plaque. In our follow-up study of 177 patients who received medical management alone, 4.0% (7/177) experienced cerebrovascular events. When considering only the CW group, a stroke recurrence rate of 18.1% (2/11) was observed in symptomatic CW patients, which is consistent with previous studies [6, 14]. It is worth mentioning that standard medical management alone may not provide sufficient protection for CW patients who have concomitant atrial fibrillation [14]. Therefore, both CEA and CAS may be considered as potentially better secondary stroke prevention strategies for CW patients [6], although further clinical trials are needed to validate the efficacy of these invasive approaches.

Several limitations need to be addressed. Firstly, the study only included patients with CW and CW with plaque who underwent CEUS and SMI examinations. This may introduce patient selection bias and result in an elevated prevalence of CW. Secondly, this study focused on the clinical and multimodal sonographic characteristics of CW and CW with plaques. Unfortunately, no cohorts of subjects with only atherosclerosis plaque were included in this study, making it impossible to compare the findings against plaque without CW subjects. However, this is an exploratory direction for our future research, and external validation is warranted. Furthermore, although 24.1% of participants had CAE, not all specimens could be obtained due to disciplinary collaboration interests and economic costs to the patients. As a result, there is a lack of histological evidence for suspected CW and CW with plaque. Lastly, the short follow-up interval for some participants may underestimate the stroke recurrence rate.

In conclusion, the multimodal ultrasonic and clinical manifestations of CW and CW with plaque are substantially different. CWs typically exhibit iso- or hypoechoic shelf-like structures projecting to the lumen with swirling blood flow on CUS, accompanied by a thin triangular endoluminal defect on the longitudinal plane on SMI, and no enhancement on CEUS. On the other hand, CW with plaque is characterized by membrane-like structures protruding from the plaque surface into the lumen or growing along the long axis of the plaque. The latter is always accompanied by internal cavity. For CW patients, the web length is an independent risk factor for luminal stenosis. However, there is no obvious correlation between CW angle, length, and symptom development. For CW with plaque patients, luminal stenosis and plaque length are risk factors for symptoms.

### Supplementary Information

**Additional file 1.**

**Additional file 2: Supplementary Movie 1.** Conventional ultrasound video of a hypoechoic carotid plaque with multiple webs which locates on the anterior wall of the left carotid artery bifurcation.

**Additional file 3: Supplementary Movie 2.** Superb microvascular imaging video of a carotid web with plaque formation which locates on the anterior wall of the left carotid artery bifurcation.

**Additional file 4: Supplementary Movie 3.** Contrast-enhanced ultrasound video of a carotid web with plaque formation which locates on the anterior wall of the left carotid artery bifurcation.

## Data Availability

Not applicable.

## Abbreviations

CAE: Carotid endarterectomy
CAS: Carotid stenting
CDFI: Color doppler flow imaging
CEUS: Contrast-enhanced ultrasound
CTA: Computed tomographic angiography
CW: Carotid web
Hcy: Homocysteine
HDL: High-density lipoprotein
HRMRI: High-resolution magnetic resonance imaging
LDL: Low-density lipoprotein
SMI: Superb microvascular imaging
TC: Total cholesterol
TG: Triglyceride
TI: Turbulence intensity
WSS: Wall shear stress
