# Supplementary material for: The differences between carotid web and carotid web with plaque: based on multimodal ultrasonic and clinical characteristics
Source: Insights Imaging. 2024 Mar 18;15:78. doi: 10.1186/s13244-024-01650-7 (PMC10948691; doi:10.1186/s13244-024-01650-7)
Supplement: Supplementary file 1 — Additional file 1. [file 13244_2024_1650_MOESM1_ESM.pdf]

The differences between carotid web and carotid web with plaque: based on multimodal ultrasonic and clinical characteristics

ELECTRONIC SUPPLEMENTARY MATERIAL

Supplementary 1

CTA imaging protocol

CT angiography was performed with two different scanners.

Scanner A: a 128-slice scanner (Brilliance 128, Philips Healthcare, Best, the Netherlands). Scanning parameters: tube voltage 120 kV, pitch 0. 984, recombination layer thickness 1. 25 mm, reassembled layer spacing 0. 625 mm, rotation time 0. 5 s.

Scanner B: 64-row CT scanner with z-flying focal-spot technology (Somatom Sensation Cardiac 64; Siemens AG; Erlangen, Germany), using the following parameters: 64 × 0.6 mm detector configuration, tube potential 100 kV, tube current modulated using the Combined Applications to Reduce Exposure (CARE) Dose 4D system (Siemens Medical Solutions, Forchheim, Germany) with a reference value of 120 mAs, pitch 1.2, gantry rotation time 0.33 s, field of view 28 cm, slice thickness 1 mm,

An 18-gauge intravenous catheter was placed in the antecubital vein; dose 1. 0 ~ 1. 2 ml /kg body weight, iomeprol 350 mg/ml (Iomeron®, Bracco, Milan, Italy), flow rate 4. 5 ml /s, and then rinse with 30 ml normal saline.

HRMRI imaging protocol

All patients were scanned on a 3-T MR scanner (MAGNETOM Verio, Siemens Healthineers) with a 16-channel head coil and an 8-channel carotid coil. The subject was placed in a supine position, with the head slightly tilted back, and the coil was fixed in the center along the angle of the bilateral mandibles; the patient kept in a comfortable position and still, and the swallowing movement and amplitude were reduced, so as to fully expose the blood vessels of the neck, and then the 3D-TOF-MRA scanning was performed on the carotid arteries of the bilateral carotid arteries to determine the location of the bifurcation of the carotid arteries.

3D-TOF-MRA was performed on both carotid arteries to determine the location of the

carotid bifurcation, and within 3 cm above and below the carotid bifurcation were subjected to T1WI (TR 800.0 ms, TE 9.3~11.0 ms, repetition chain length 8, acquisition time 8 min, a total of 12 levels), T2WI (TR 2,500~3,700 ms, repetition chain length 8, acquisition time 8 min, a total of 12 levels), 3D-TOF (TR 23.0 ms, TE 3.5 ms, flip angle 25°, acquisition time 3 min, total 40 layers), and three-dimensional magnetized intensity preparatory gradient echo sequence (3D MP-RAGE) (TR 8.8 ms, TE 5.3 ms, acquisition time 3 min, total 40 layers).The total scanning time was approximately 40 min.

The time interval between clinical laboratory test data and imaging data was within two weeks.

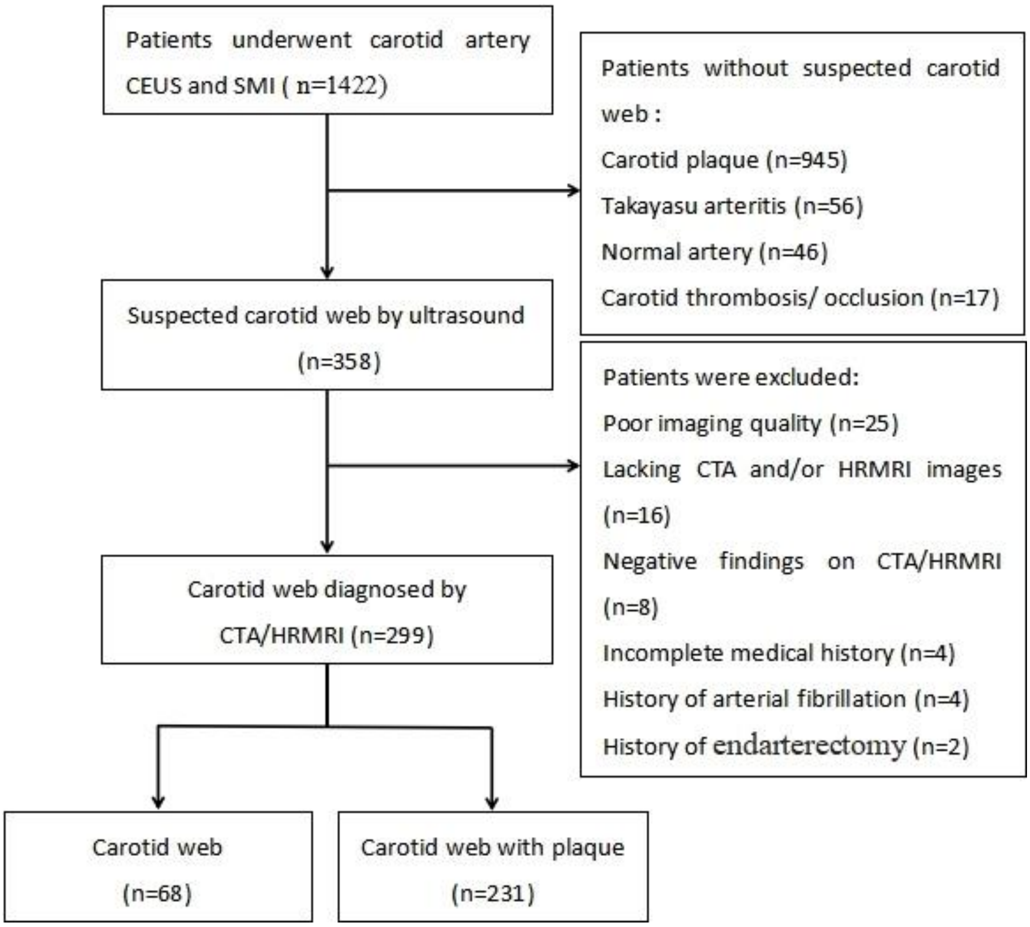

Supplementary Figure 1 The flowchart diagram of patient selection.

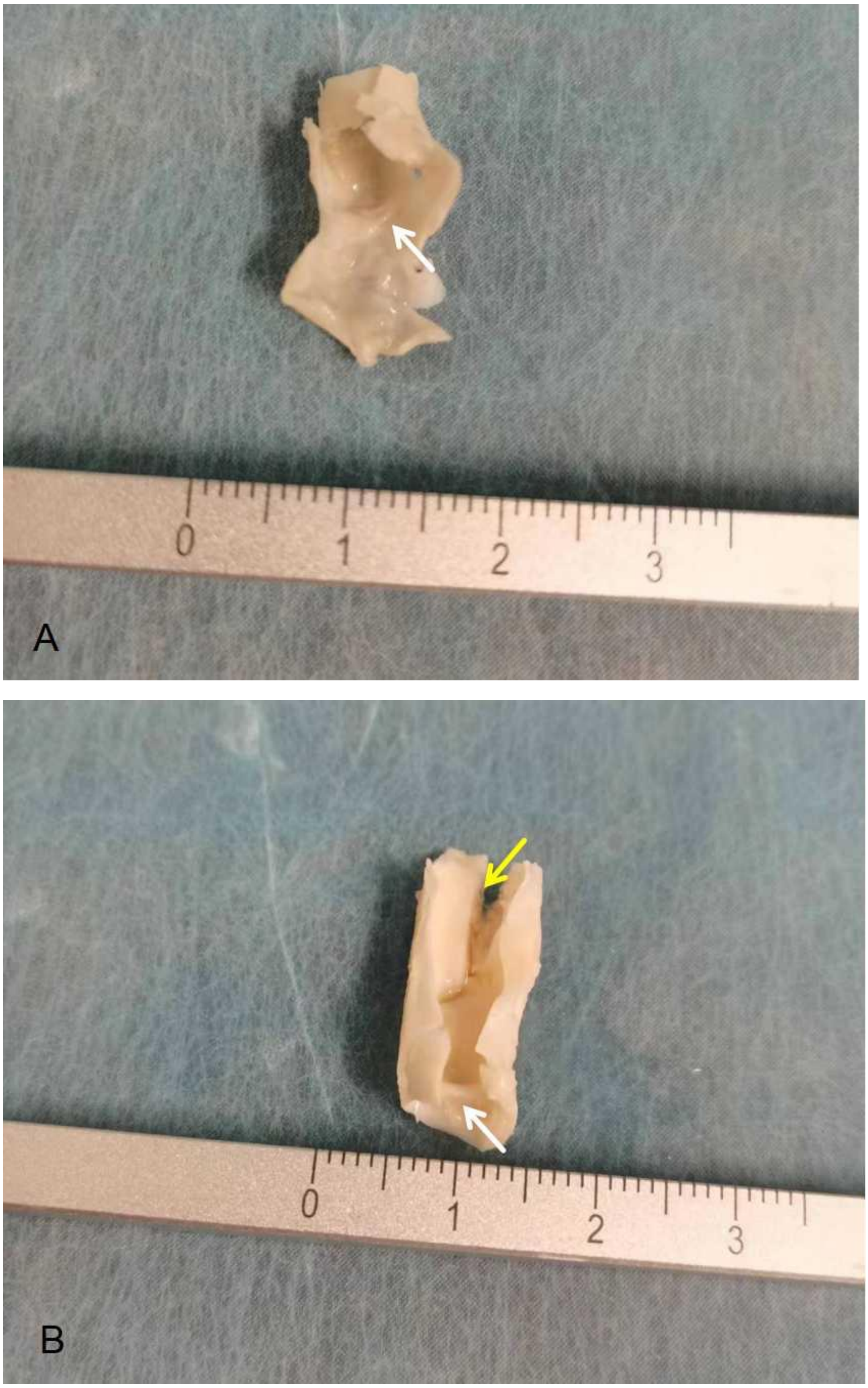

Supplementary Figure 2 Surgical specimens of carotid web (**A**, white arrow) and carotid web with plaque (**B**, white arrow indicates web, yellow arrow indicates plaque).

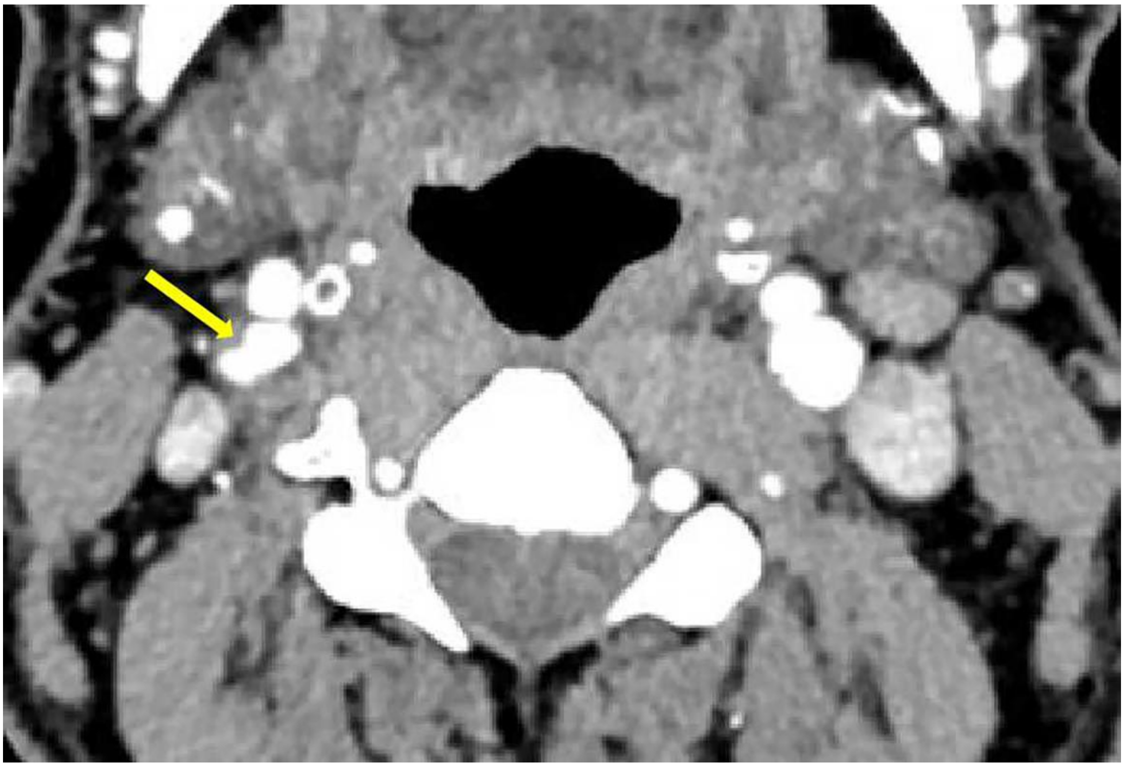

Supplementary Figure 3 CTA image of a carotid web (yellow arrow) on the anterior wall of the right carotid artery bifurcation.

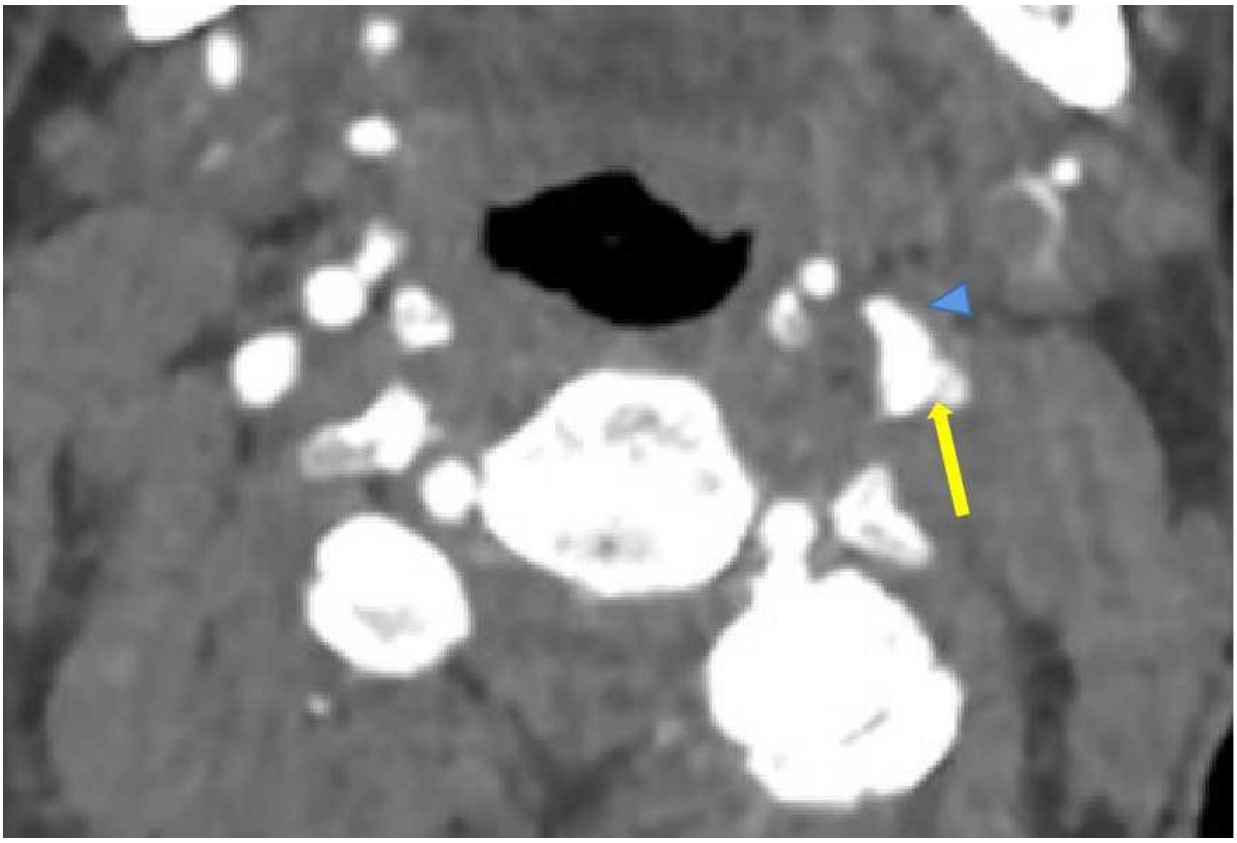

Supplementary Figure 4 CTA image of a carotid web (yellow arrow) with plaque (blue arrowhead) on the anterior wall of the left carotid artery bifurcation.

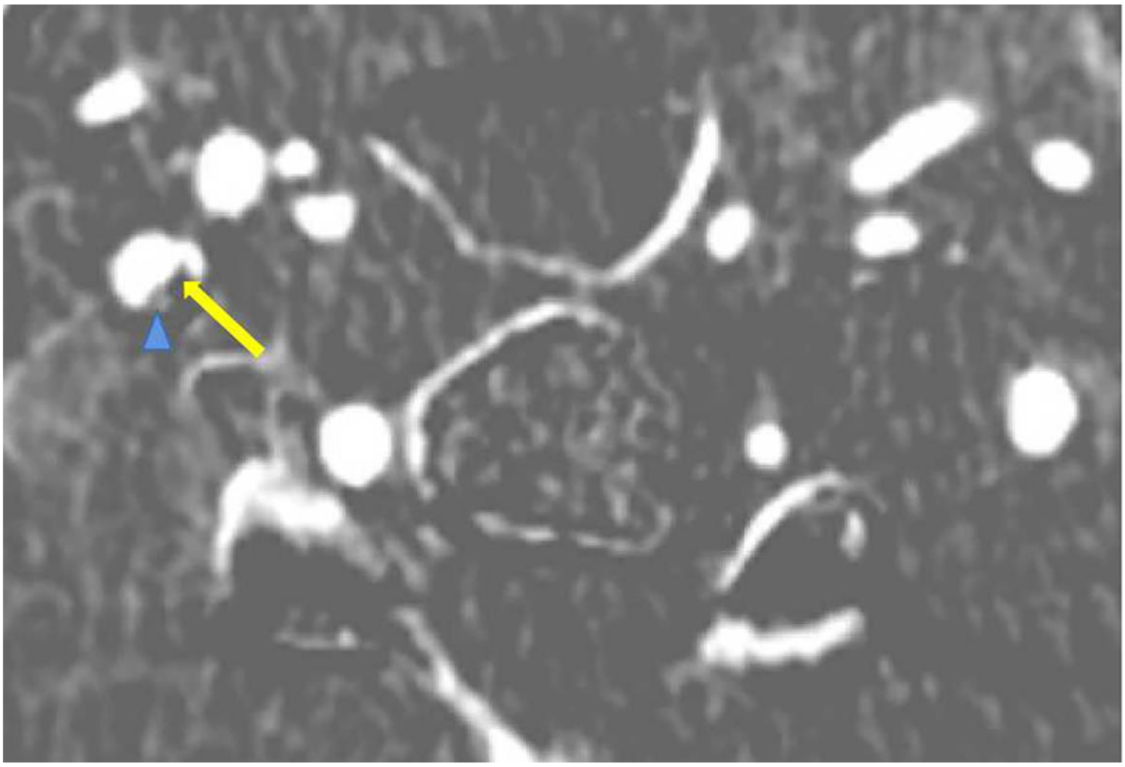

Supplementary Figure 5 CTA image of a carotid web (yellow arrow) with plaque (blue arrowhead) on the anterior wall of the right carotid artery bifurcation.

Supplementary Table 1: Logistical analysis of potential risk factors for symptom caused by CW or CW with plaque

Variables	Univariate analysis				Multivariate analysis			
	OR	95% CI		*P* value	OR	95% CI		*P* value
		Lower bound	Upper bound			Lower bound	Upper bound	
**CW**								
luminal stenosis	0.327	0.064	1.658	0.177				
web length	1.087	0.844	1.399	0.875				
web thickness	0.879	0.223	3.467	0.519				
web angle	1.002	0.977	1.028	0.854				
**CW with plaque**								
luminal stenosis	1.929	1.0831	3.436	0.026	1.860	1.021	3.391	0.043
web length	0.995	0.897	1.104	0.924				
web thickness	0.977	0.666	1.433	0.905				
web angle	1.0	0.991	1.009	0.949				
plaque length	0.947	0.912	0.985	0.006	0.955	0.918	1.014	0.023
plaque thickness	0.843	0.101	0.688	1.034				
plaque enhancement	1.340	0.672	2.670	0.406				
age	0.965	0.929	1.003	0.068	0.974	0.936	1.014	0.205

Notes: CW, carotid web; OR: odds ratio; CI: confidence index

Supplementary Table 2: Logistical analysis of potential risk factors for luminal stenosis caused by CW or CW with plaque

Variables	Univariate analysis				Multivariate analysis			
	OR	95% CI		*P* value	OR	95% CI		*P* value
		lower bound	upper bound			lower bound	upper bound	
**CW**								
web length	1.653	1.139	2.399	0.008	1.653	1.139	2.399	0.008
web thickness	1.809	0.395	8.288	0.445				
web angle	0.99	0.942	1.038	0.647				
**CW with plaque**								
web length	1.040	0.942	1.149	0.436				
web thickness	1.799	1.105	2.930	0.018	1.870	1.099	3.182	0.021
web angle	1.006	0.997	1.016	0.179				
plaque length	1.034	0.998	1.072	0.065	0.995	0.955	1.037	0.815
plaque thickness	1.682	1.310	2.160	<0.001	1.587	1.199	2.101	0.001
plaque enhancement	0.436	0.230	0.824	0. 011	0.370	0.177	0.773	0.008
age	1.023	0.988	1.059	0. 193				

Notes: CW, carotid web; OR: odds ratio; CI: confidence index
